# Mitochondrial function in immature bovine oocytes is improved by an increase of cellular cyclic AMP

**DOI:** 10.1038/s41598-019-41610-6

**Published:** 2019-03-26

**Authors:** Shu Hashimoto, Masaya Yamanaka, Takayuki Yamochi, Hisataka Iwata, Ryouka Kawahara-Miki, Masayasu Inoue, Yoshiharu Morimoto

**Affiliations:** 1 Graduate School of Medicine, Osaka City University, Osaka, 545-8585 Japan; 2 IVF Namba Clinic, Osaka, 550-0015 Japan; 3 Department of Animal Science, Tokyo University of Agriculture, Kanagawa, 243-0034 Japan; 4 Nodai Genome Research Center, Tokyo University of Agriculture, Tokyo, 156-8502 Japan; 5 HORAC Grandfront Osaka Clinic, Osaka, 530-0011 Japan

## Abstract

Although *in vitro* maturation (IVM) of oocytes is important for assisted reproduction, the rate of development of embryos from IVM oocytes is lower than from their *in vivo* counterparts. It has been shown that an artificial increase of intracellular cAMP before culture significantly improves oocyte developmental competence in cattle and mice. Here, we revealed that forskolin and 3-isobutyl-1-methylxanthine treatment of prophase-stage oocytes induced the expression of genes required for glycolysis, fatty acid degradation, and the mitochondrial electron transport system and improved mitochondrial functions and ATP levels in oocytes without involving nuclear maturation. We propose the existence of a comprehensive energy-supply system in oocytes under follicle-stimulating hormone stimulation as a potential explanation of how oocytes acquire developmental competence.

## Introduction

*In vitro* maturation (IVM) of oocytes is an important technology for assisted reproduction (ART), having a wide range of research and clinical applications. IVM is usually used to generate mature oocytes not only for human infertility treatment^1^ but also for animal reproduction. However, it is generally accepted that the developmental competence of embryos produced using IVM oocytes is lower than that of their *in vivo* counterparts, probably owing to an inappropriate status of the cytoplasm^2,3^.

Meiotic maturation resumes immediately after the isolation of oocytes from follicles. It has been speculated that this rapid resumption of meiosis (spontaneous maturation) precludes the achievement of developmental competence by oocytes. A high concentration of cAMP in oocytes is involved in their meiotic arrest^4,5^. Several pathways are involved in cAMP signaling, which communicate through sophisticated crosstalk. Mainly, cAMP propels protein kinase A (PKA)^6^, which regulates the activity of Cyclin-dependent kinase 1 (CDK1) involved in the inhibition of oocyte maturation^7^. In addition, cAMP communicates with the cGMP pathway. The cGMP from granulosa cells transfers through gap junctions and upholds high cAMP levels by inhibiting phosphodiesterase (PDE) 3A in oocytes^8^. These findings suggest that cAMP activates various pathways to inhibit meiotic resumption. Activation of PKA results in the stimulation of key transcription factors including SP1/SP3 and CREB, which eventually control the transcription of downstream genes involved in cellular growth, steroidogenesis, and morphogenesis^9^.

The downstream pathways through which cAMP tunes meiotic maturation have not been fully characterized, except for the inhibition of meiotic resumption in oocytes^4,5^. It has been shown that an artificial increase of intracellular cAMP during oocyte maturation significantly promotes oocyte developmental ability in cattle and mice^10–12^. Widely, high values of intracellular cAMP are maintained either by phosphodiesterase (PDE) inhibitors, such as 3-isobutyl-1-methylxanthine (IBMX), to halt cAMP degradation or through continuous activation of adenylate cyclase (AC) with agents such as forskolin (FSK)^12^. It has been supposed that, during *in vitro* culture, prevention of an early resumption of oocyte maturation through cAMP modulation inhibits the activation of maturation-promoting factor (MPF)^13^ and partially compensates for the constrained oocyte developmental ability. However, the precise mechanism by which cAMP betters oocyte competence during an increase of cAMP remains to be elucidated. The present work describes the changes of gene expression in oocytes and surrounding granulosa cells following FSK and IBMX treatment, as determined by transcriptome analyses, and the corresponding improvement of these oocytes’ mitochondrial function.

## Results

### cAMP contents of oocytes

FSK and IBMX treatment during 2 hours increased the cAMP content of immature oocytes (P < 0.001; 462.17 ± 45.32 (mean ± SD) fmol/oocyte, N: 120) compared with that of oocytes immediately after their retrieval from 2–5-mm follicles (0.37 ± 0.22 fmol/oocyte, N: 400).

### Transcriptome analysis

The transcriptome data have been deposited in the DDBJ Sequence Read Archive (DRA) with accession number DRA006403. In comparing the transcriptomes between FSK-IBMX treatment and control, 1,125 genes were differentially expressed in oocytes (P < 0.01, |fold changes| > 2) and 2,693 genes were differentially expressed in granulosa cells (P < 0.01, |fold changes| > 10). Among these differentially-expressed genes, 1,116 genes were upregulated and 9 genes were down-regulated in oocytes. Four hundred one genes were upregulated and 2,292 genes were down-regulated in granulosa cells.

### Meiotic maturation

**Figure 1:**
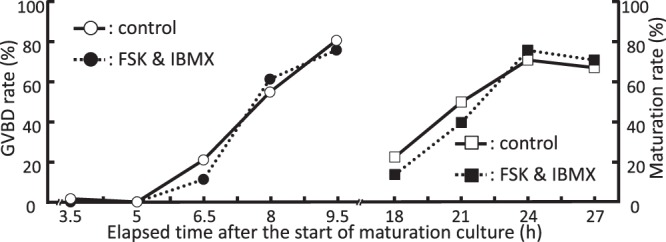
Meiotic maturation following forskolin (FSK) and 3-isobutyl-1-methylxanthine (IBMX) treatment. Change in nuclear status over time was assessed following FSK and IBMX treatment. During *in vitro* maturation, 20 oocytes per treatment from 3 independent experiments were fixed at several time points and stained to assess the time-dependent change of nuclear maturation. A total of 1080 oocytes were examined. Time 0 represents the start of maturation culture. Open circle: germinal vesicle breakdown (GVBD) in control, closed circle: GVBD following FSK and IBMX treatment, open square: metaphase II oocytes in control, closed square: metaphase II oocytes following FSK and IBMX treatment.

Although the expression of several genes related to the progression of meiotic maturation in oocytes was upregulated after FSK and IBMX treatment, *Wee1* which inhibits the entry in the metaphase and results in cell cycle arrest was drastically upregulated and the key gene for MPF activation, *Cdc*2*5* (*Cdc*2*5a* and *Cdc25b*), was downregulated in oocytes (Table 1). The duration required for meiotic maturation of oocytes after FSK and IBMX treatment was the same as that of control oocytes (Fig. 1), indicating that the progression of the cell cycle was arrested during the treatment. Thus, we compared gene expression immediately after oocyte pick-up and after FSK and IBMX treatment in oocytes and granulosa cells. The findings from this comparison revealed that histone *H2A* variants, *H2AFY* and *H2AFY2*, were upregulated after FSK and IBMX treatment.

**Table 1:** Change of expression of genes related to meiotic resumption in oocytes following FSK and IBMX treatment.

Feature ID	Annotations - database object name	Experiment –fold change* (original values)	Kal’s Z-test: P-value correction	Number of mapped reads before FSK and IBMX treatment	Number of mapped reads after FSK and IBMX treatment	Change of gene expression
**WEE1**	**WEE1 G2 checkpoint kinase**	**9.7692308**	**0**	**13**	**127**	**upregulated**
**H2AFY2**	**Histone H2A (macroH2A2)**	**8**	**0.006904653**	**2**	**16**	**upregulated**
**H2AFY**	**Histone H2A (macroH2A1)**	**1.2139303**	**0.010222819**	**603**	**732**	**upregulated**
**ARPP19**	**cAMP-regulated phosphoprotein 19**	**1.2087407**	**2.60E-06**	**1,739**	**2,102**	**upregulated**
**CKS1B**	**Cyclin-dependent kinase regulatory subunit 1**	**1.1900074**	**0**	**8,126**	**9,670**	**upregulated**
**PTTG1**	**Securin**	**1.1384134**	**0**	**37,099**	**42,234**	**upregulated**
**CDK1**	**Cyclin-dependent kinase 1**	**1.1268339**	**0.006824813**	**2,113**	**2,381**	**upregulated**
RB1	Retinoblastoma-associated protein	1.0946502	0.853703543	243	266	unchanged
CDC5L	Cell division cycle 5-like protein	1.0925637	0.136283354	1,923	2,101	unchanged
**CCNB2**	**G2/mitotic-specific cyclin-B2**	**1.088404**	**3.04E-05**	**10,633**	**11,573**	**upregulated**
**CDC25C**	**M-phase inducer phosphatase 3**	**1.0860763**	**0.000989742**	**7,656**	**8,315**	**upregulated**
**C-MOS**	**Proto-oncogene serine/threonine-protein kinase mos**	**1.0601145**	**0.006690682**	**15,720**	**16,665**	**upregulated**
ENSA	Alpha-endosulfine	1.0523823	0.123577445	10,767	11,331	unchanged
CCNB1	G2/mitotic-specific cyclin-B1	1.0378686	0.428368701	17,772	18,445	unchanged
MASTL	Serine/threonine-protein kinase greatwall	1.0317363	1	3,277	3,381	unchanged
*CDC25A*	*M-phase inducer phosphatase 1*	*−1.0940605*	*3.20E-07*	*5,176*	*4,731*	*downregulated*
*PKMYT1*	*Protein kinase, membrane-associated tyrosine/threonine 1*	*−1.1303*	*0.0007733*	*1,535*	*1,358*	*downregulated*
*CDC25B*	*M-phase inducer phosphatase 2*	*−1.1393222*	*0*	*9,715*	*8,527*	*downregulated*

*When the ratio is less than 1, it is converted to its negative inverse.

The transcriptome data have been deposited in the DDBJ Sequence Read Archive (DRA) with accession number DRA006403.

Bold show upregulated. Italic show downregulated.

### Developmental competence to the blastocyst stage

FSK and IBMX treatment increased the developmental rate to the blastocysts stage on day 7 after fertilization (P < 0.01 by chi-square test, 27.5% (33/120)) compared with that of control oocytes (16.7% (20/120)). There were no differences in the maturation (FSK and IBMX: 75% (15/60) vs. control: 81% (47/58)), the normal fertilization (FSK and IBMX: 76.4% (42/55) vs. control: 69.1% (38/55)), and the blastocyst formation rates on day 8 after insemination (FSK and IBMX: 33.3% (40/120) vs. control: 25% (30/120)). The cell number of blastocysts on day 8 of oocytes treated with FSK and IBMX (168.4 ± 33.6, N: 33) was similar to that of control oocytes (157 ± 3.62, N: 21).

### Mitochondrial function in oocytes

**Figure 2:**
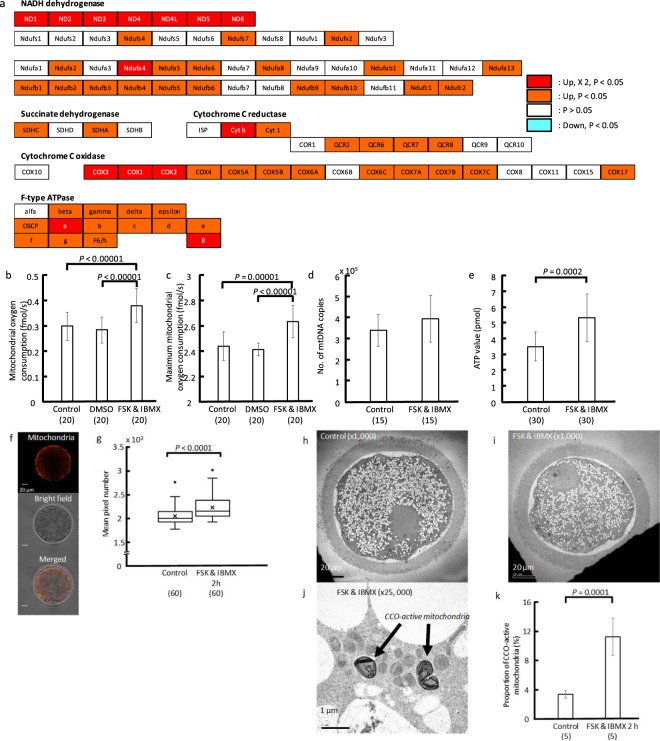
Change of expression of genes involved in mitochondrial function in oocytes following FSK and IBMX treatment. (**a**) Each box shows the protein constituents of mitochondrial complex, NADH dehydrogenase, succinate dehydrogenase, cytochrome C reductase, cytochrome C oxidase, and ATPase. Red and orange boxes indicate that gene expression is significantly upregulated (P < 0.05). White and blue boxes show that gene expression is unchanged and downregulated, respectively. Red box also means that the expression level is increased more than double in oocytes after FSK and IBMX treatment compared with control oocytes. This was produced in reference to KEGG pathway for oxidative phosphorylation in Bos Taurus (https://www.genome.jp/kegg-bin/show_pathway?org_name=bta&mapno=00190&mapscale=&show_description=show). (**b**) Bar chart shows the oxygen consumption in mitochondria in oocytes following FSK and IBMX treatment for 2 h. Mitochondrial oxygen consumption significantly increased upon FSK and IBMX treatment (P < 0.00001). (**c**) Bar chart shows the maximum oxygen consumption in mitochondria in oocytes following FSK and IBMX treatment for 2 h. Maximum mitochondrial oxygen consumption significantly increased upon FSK and IBMX treatment (P < 0.0001). (**d**) Bar chart shows the mitochondrial DNA (mtDNA) copy number in oocytes following FSK and IBMX treatment for 2 h, as determined using real-time PCR. The copy number of mtDNA in oocytes did not change. (**e**) Bar chart shows ATP levels in oocytes following FSK and IBMX treatment for 2 h, measured as luminescence generated in an ATP-dependent luciferin–luciferase bioluminescence assay. ATP level in oocytes significantly increased after treatment (P < 0.001). (**f**) Representative image of MitoTracker-stained oocyte. (**g**) The boxplot shows active mitochondria in oocytes (mean pixel number). FSK and IBMX treatment significantly increased active mitochondria in oocytes (P < 0.0001). Parentheses show the numbers of oocytes examined. Data were compared using *t*-test. (**h–j**) Representative ultrastructures in oocytes before and after FSK and IBMX treatment. (**h,i**): x1,000; (**j**): x25,000. Black arrows show mitochondria with cytochrome C oxidase activity. (**k**) Bar chart shows the proportion of mitochondria with cytochrome C oxidase activity in oocytes following FSK and IBMX treatment for 2 h. FSK and IBMX treatment significantly increased the proportion of mitochondria with cytochrome C oxidase activity (P < 0.001). Parentheses show the numbers of oocytes examined. Data in 2b and c were obtained from the same samples. Oxygen consumption was measured using scanning electrochemical microscopy. Data were compared by *t*-test or ANOVA followed by the Tukey–Kramer test.

Increase of intracellular cAMP induced a cAMP-dependent gene expression pattern (DRA006403). In particular, the expression of genes encoding proteins involved in a substantial proportion of mitochondrial functions in oxidative phosphorylation was significantly increased following FSK and IBMX treatment (Fig. 2a). The oxygen consumption rates (OCRs) and maximum OCRs (maxOCRs) in the treated oocytes immediately after treatment (0.38 ± 0.07 and 2.63 ± 0.13 fmol/s, N: 20, respectively; Fig. 2b,c) significantly increased (P < 0.0001) compared with those of control oocytes (0.30 ± 0.06 and 2.44 ± 0.11 fmol/s, N: 20, respectively) and of oocytes cultured in 2% DMSO for 2 h (0.28 ± 0.05 and 2.41 ± 0.05 fmol/s, N: 20, respectively). However, the copy number of mitochondrial DNA (mtDNA) remained unchanged (FSK and IBMX treatment (N: 15): 393,491 ± 111,456 vs. control (N: 15): 339,679 ± 76,316, Fig. 2d). The level of ATP in the treated oocytes immediately after treatment (5.34 ± 1.5 pmol, N: 30, Fig. 2e) was also significantly increased (P < 0.001) compared with that of control oocytes (3.51 ± 0.92 pmol, N: 30). Moreover, the mitochondrial activity (FSK and IBMX treatment (N: 60): 2,229 ± 267 pixels vs. control (N: 60): 2,048 ± 185 pixels, Fig. 2f,g) and the incidence of cytochrome C oxidase (CCO)-active mitochondria (FSK and IBMX treatment (N: 5): 11.3 ± 2.5% vs. control (N: 5): 3.4 ± 0.5%, Fig. 2h–k) significantly increased (both P < 0.0001) in oocytes after treatment.

### Beta-oxidation in oocytes

**Figure 3:**
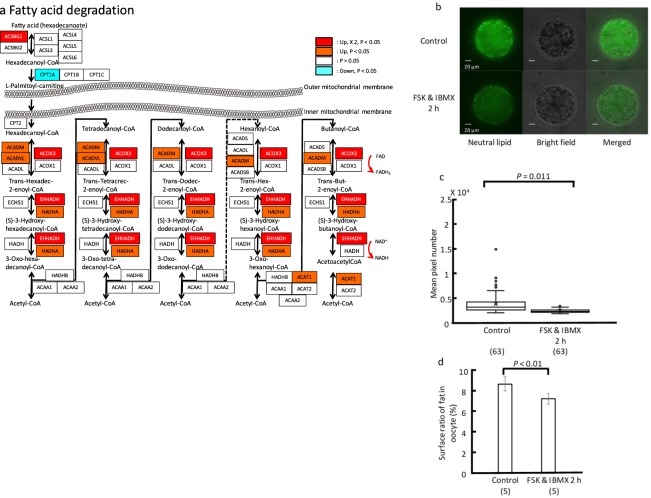
Change of expression of genes involved in fatty acid degradation in oocytes following FSK and IBMX treatment. (**a**) Schema of beta-oxidation in oocytes. Each box shows enzymes involved in beta-oxidation. Red and orange boxes indicate that gene expression is significantly upregulated (P < 0.05). White and blue boxes show that gene expression is unchanged and downregulated, respectively. Red box also means that the expression level is increased more than double in oocytes after FSK and IBMX treatment compared with control oocytes. (**b**) Representative images of neutral lipids in oocytes. Upper panel: Oocytes were fixed immediately after oocyte pick-up and stained with BODIPY™ 493/503. Lower panel: Oocytes were fixed after FSK and IBMX treatment for 2 h. (**c**) The boxplot shows neutral lipid content in oocytes (mean pixel number). FSK and IBMX treatment significantly decreased neutral lipid content in oocytes. (**d**) The bar chart shows the area ratio occupied by lipids in cytoplasm. Data were compared by *t*-test. Parentheses show the numbers of oocytes examined. *ACSBG1* and *ACSBG2*: acyl-CoA synthetase bubblegum family members 1 and 2, respectively. *ACSL1–6*: acyl-CoA synthetase long-chain family members 1–6, respectively. *CPT1A*, *1B*, *1C*, and *2*: carnitine palmitoyltransferases 1A, 1B, 1C, and 2, respectively. *ACADVL*, *ACADL*, *ACADM*, *ACADS*, and *ACADSB*: acyl-CoA dehydrogenases, very long chain, long chain, C-4 to C-12 straight chain, C-2 to C-3 short chain, and short/branched chain, respectively. *ACO1* and *ACOX3*: acyl-CoA oxidases 1 and 3, respectively. *ECHS1*, *HADHA*, and *EHHADH*: enoyl-CoA hydratase, short chain 1; hydroxyacyl-CoA dehydrogenase/3-ketoacyl-CoA thiolase/enoyl-CoA hydratase (trifunctional protein), alpha subunit; and enoyl-CoA hydratase and 3-hydroxyacyl CoA dehydrogenase. *HADH*: hydroxyacyl-CoA dehydrogenase. *HADHB*, *ACAA1*, and *ACAA2*: hydroxyacyl-CoA dehydrogenase/3-ketoacyl-CoA thiolase/enoyl-CoA hydratase (trifunctional protein), beta subunit; acetyl-CoA acyltransferase 1; and acetyl-CoA acyltransferase 2. *ACAT1* and *ACAT2*: acetyl-CoA acetyltransferase 1 and 2, respectively.

We assessed the expression of genes involved in beta-oxidation, which is one of the suppliers of the electron donors NADH and FADH_2_ to examine whether fatty acid degradation is involved in the improvement of mitochondria function. FSK and IBMX treatment did not affect the expression of lipase, which hydrolyzes triglycerides into their component fatty acids and glycerol. However, the expression of lipase maturation factor 1 (*LMF 1*), *LMF 2*, and lipoprotein lipase (*LPL*) was significantly upregulated (P < 0.001, Table 2). Because *LMF 1* is an endoplasmic reticulum (ER) membrane protein involved in the post-translational folding and/or assembly of *LPL* and hepatic lipase (*HL*) into active enzymes^14^, the upregulation of *LMF 1*, *LMF 2*, and *LPL* indicated that lipid hydrolysis was occurring. In addition, the expression of scavenger receptor class B member 1 (*SCARB1*), which functions as a receptor for high-density lipoprotein, low-density lipoprotein receptor (*LDLR*), solute carrier family 27 (fatty acid transporter), member 1 (*SLC27A1*) and member 3 (*SLC27A3*), and fatty acid binding protein (*FABP3*), which plays an important role in the transportation of fatty acids from granulosa cells to oocytes^15^, was also upregulated in oocytes after FSK and BMX treatment (P < 0.001), suggesting the facilitation of transport of fatty acids between cellular membranes^16^. Although the expression of carnitine-palmitoyltransferase 1 (*Cpt1*) was slightly downregulated, that of carnitine-palmitoyltransferase 2 (*Cpt2*) remained unchanged. Moreover, the expression of long-chain acyl-CoA ligase ACSBG (*ACSBG1*: EC 6.2.1.3), which activates the breakdown of complex fatty acids, acyl-CoA dehydrogenase (*ACADM*: EC 1.3.8.7), and very long chain acyl-CoA dehydrogenase (*ACADVL*: EC 1.3.8.9), which function to catalyze the initial step in each cycle of fatty acid β-oxidation in the mitochondria, as well as enoyl-CoA hydratase (*HADHA* and *EHHAD*: EC 4.2.1.17), was significantly upregulated in oocytes after FSK and IBMX treatment (Fig. 3a), suggesting the promotion of β-oxidation in the mitochondria and increases of NADH, FADH_2_, and acetyl-CoA. Finally, lipid content in oocytes decreased following FSK and IBMX treatment (P < 0.01, FSK and IBMX treatment (N: 63): 2,533.9 ± 343.3 vs. control (N: 63): 3,972.2 ± 2,161.4 pixels in Fig. 3b,c; FSK and IBMX treatment (N: 5): 7.2 ± 0.48 vs. control (N: 5): 9.64 ± 0.09% in Fig. 3d).

**Table 2:** Change of expression of genes involved in fatty acid degradation in oocytes following FSK and IBMX treatment.

Feature ID	Annotations –database object name	Experiment – fold Change* (original values)	Kal’s Z-test: P-value correction	Number of mapped reads before FSK and IBMX treatment	Number of mapped reads after FSK and IBMX treatment	Change of gene expression
**LPL**	**Lipoprotein lipase**	**27.666667**	**0**	**3**	**83**	**upregulated**
**LMF1**	**Lipase maturation factor 1**	**16.5**	**0**	**6**	**99**	**upregulated**
**SCARB1**	**Scavenger receptor class B member 1**	**8.2444444**	**0**	**45**	**371**	**upregulated**
**SLC27A3**	**Solute carrier family 27 (fatty acid transporter), member 3**	**3.8367347**	**0**	**98**	**376**	**upregulated**
**LMF2**	**Lipase maturation factor 2**	**3.0294118**	**1.08E-07**	**34**	**103**	**upregulated**
**LDLR**	**Low-density lipoprotein receptor**	**1.973822**	**0**	**382**	**754**	**upregulated**
LIPJ	Lipase	1.8333333	0.307338882	12	22	unchanged
**SLC27A1**	**Solute carrier family 27 (fatty acid transporter), member 1**	**1.6265487**	**0**	**565**	**919**	**upregulated**
**FABP3**	**Fatty acid-binding protein, heart**	**1.1520605**	**7.40E-07**	**3,834**	**4,417**	**upregulated**
LIPA	Lipase	1.0908747	0.070295659	2,641	2,881	unchanged
CD36	Platelet glycoprotein 4	1.0801887	0.818061605	424	458	unchanged
FABP5	Fatty acid-binding protein, epidermal	1.059115	0.472063977	2,825	2,992	unchanged
FABP7	Fatty acid-binding protein, brain	−1.6	0.774294053	8	5	unchanged
LIPM	Lipase	#DIV/0!	0.685944354	1	0	unchanged

*When the ratio is less than 1, it is converted to its negative inverse.

The transcriptome data have been deposited in the DDBJ Sequence Read Archive (DRA) with accession number DRA006403.

Bold show upregulated.

### Glycolysis in oocytes

**Figure 4:**
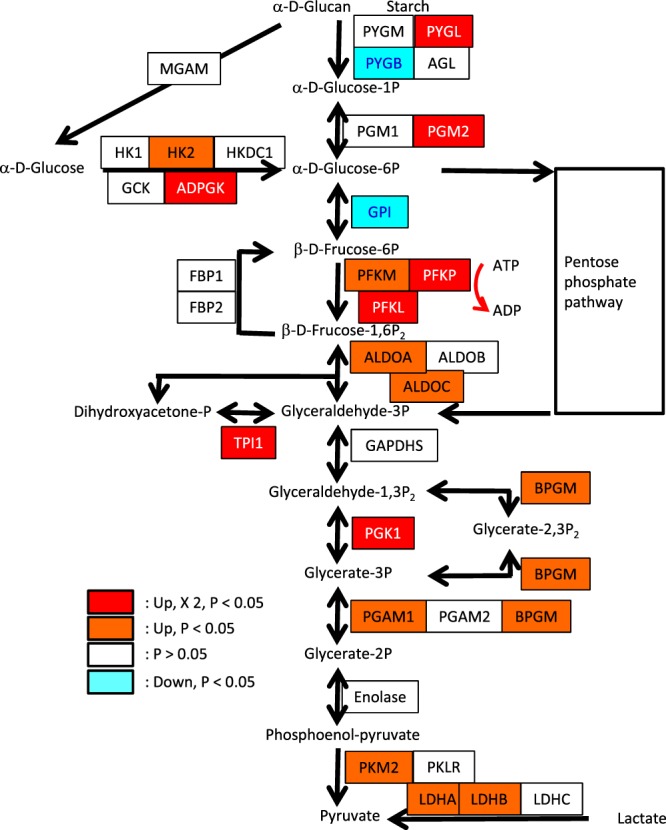
Schema of expression of genes involved in glycolysis in oocytes following FSK and IBMX treatment. Each box shows enzymes involved in glycolysis. Red and orange boxes indicate that gene expression is significantly upregulated (P < 0.05). White and blue boxes show that gene expression is unchanged and downregulated, respectively. Red box also means that the expression level is increased more than double in oocytes after FSK and IBMX treatment compared with control oocytes. *PYGL*, *PYGM*, and *PYGB*: glycogen phosphorylases; *AGL*: glycogen debranching enzyme; *MGAM*: maltase-glucoamylase; *PGM1*: phosphoglucomutase-1; *PGM2*: phosphoglucomutase-2; *HK1*, *HK2*, and *HKDC1*: hexokinases; *GCK*: glucokinase; *ADPGK*: ADP-dependent glucokinase; *GPI*: glucose-6-phosphate isomerase; *PFKM*, *PFKL*, and *PFKP*: 6-phosphofructokinases 1; *FBP1* and *FBP2*: fructose-1,6-bisphosphatases I; *ALDOA*, *ALDOB*, and *ALDOC*: fructose-bisphosphate aldolases, class I; *GAPDH*: glyceraldehyde 3-phosphate dehydrogenase; *BPGM*: bisphosphoglycerate/phosphoglycerate mutase; *PGK1*: phosphoglycerate kinase; *PGAM1* and *PAM2*: 2,3-bisphosphoglycerate-dependent phosphoglycerate mutases; *TPI1*: triosephosphate isomerase; *PKM*: pyruvate kinase; *PKLR*: pyruvate kinase isozymes R/L; *LDHA*, *LDHB*: lactate dehydrogenase. *HK2*, *ADPGK*, *PFKL*, *PFKM*, *PFKP*, *ALDOA*, *ALDOC*, and *PKM2* are rate-limiting enzymes in the glycolytic system.

The genes involved in glycolysis were also examined to assess the glycolysis involves in the improvement of mitochondria function. Several genes encoding members of the solute carrier family 2 (*SLC2A*) were upregulated when oocytes were treated with FSK and IBMX (Table 3). Hexokinase (*HK2*: EC2.7.1.1), ADP-dependent glucokinase (*ADPGK*: EC 2.7.1.147), 6-phosphofructokinase 1 (*PFKL*, *PFKM*, *PFKP*: EC 2.7.1.11), fructose-bisphosphate aldolase (*ALDOA*, *ALDOC*: EC 4.1.2.13), and pyruvate kinase (*PKM2*: EC 2.7.1.40), which are rate-limiting enzymes in glycolysis, were significantly upregulated following FSK and IBMX treatment, suggesting the increase of glycolytic activity (Fig. 4).

**Table 3:** Change of expression of solute carrier family 2 (SLC2A) genes in oocytes following FSK and IBMX treatment.

FeatureID	Annotations –database object name	Experiment –fold change* (original values)	Kal’s Z-test: P-value correction	Number of mapped reads before FSK and IBMX treatment	Number of mapped reads after FSK and IBMX treatment	Change of gene expression
**SLC2A3**	**Solute carrier family 2, facilitated glucose transporter member 3**	**14.822526**	**0**	**293**	**4343**	**upregulated**
**SLC2A12**	**Solute carrier family 2, facilitated glucose transporter member 12**	**11**	**0.022863918**	**1**	**11**	**upregulated**
**SLC2A10**	**Solute carrier family 2, facilitated glucose transporter member 10**	**9.5**	**0.001772048**	**2**	**19**	**upregulated**
**SLC2A1**	**Solute carrier family 2, facilitated glucose transporter member 1**	**4.3222222**	**0**	**270**	**1167**	**upregulated**
SLC2A5	Solute carrier family 2, facilitated glucose transporter member 5	2	1	1	2	unchanged
SLC2A4	Solute carrier family 2, facilitated glucose transporter member 4	1.5	1	2	3	unchanged
SLC2A13	Solute carrier family 2, facilitated glucose transporter member 13	1.5	0.714956314	10	15	unchanged
SLC2A8	Solute carrier family 2, facilitated glucose transporter member 8	1.0643029	0.587908435	1664	1771	unchanged
SLC2A2	Solute carrier family 2, facilitated glucose transporter member 2	−1	1	1	1	unchanged

*When the ratio is less than 1, it is converted to its negative inverse.

The transcriptome data have been deposited in the DDBJ Sequence Read Archive (DRA) with accession number DRA006403.

Bold show upregulated.

### Methyltransferase in oocytes

To assess whether FSK and IBMX affect gene expression in oocytes, the genes involved in methylation control of DNA or histone were investigated. S-Adenosylmethionine synthases (*MAT1A* and *MAT2A*, EC 2.5.1.6), which synthesize methyl-group donor and adenosylhomocysteinase (*AHCYL*, EC.3.3.1.1), were significantly upregulated in oocytes after FSK and IBMX treatment (P < 0.001, Table 4). However, the expression of genes involved in the methylation of DNA and histone proteins in immature oocytes was not upregulated. The expression of methyltransferase to histone or DNA [DNA (cytosine-5)-methyltransferase 1: *DNMT1*; 3B: *DNMT3B*; histone-lysine N-methyltransferase: *UV39H1*; histone-lysine N-methyltransferase SUV39H2: *SUV39H2*] was downregulated in oocytes after FSK and IBMX treatment. Moreover, the expression of genes involved in the transmethylation cycle remained unchanged or downregulated. Thus, the methylation of DNA and histones might remain unchanged during the treatment, suggesting that mRNAs would be accumulated in oocytes to support the development.

**Table 4:** Change of expression of genes related to methylation in oocytes following FSK and IBMX treatment.

Feature ID	Annotations – database object name	Experiment – fold change* (original values)	Kal’s Z-test: P-value correction	Number of mapped reads before FSK and IBMX treatment	Number of mapped reads after FSK and IBMX treatment	Change of gene expression
**MAT1A**	**S-Adenosylmethionine synthase**	**7.4074074**	**0**	**27**	**200**	**upregulated**
BHMT2	Betaine-homocysteine S-methyltransferase 2	1.8	0.685944354	5	9	unchanged
**MAT2A**	**S-Adenosylmethionine synthase**	**1.3667011**	**0**	**2,907**	**3,973**	**upregulated**
BHMT	Betaine-homocysteine S-methyltransferase 1	1.2213115	0.416117351	122	149	unchanged
AHCYL1	Adenosylhomocysteinase like 1	1.1280168	0.108837606	953	1,075	unchanged
AHCY	Adenosylhomocysteinase	1.0471098	0.66715386	3,927	4,112	unchanged
MAT2B	Methionine adenosyltransferase 2 subunit beta	1.0045749	0.462293673	12,022	12,077	unchanged
DNMT3L	DNA (cytosine-5)-methyltransferase 3-like	1	1	0	0	unchanged
SUV39H2	Histone-lysine N-methyltransferase SUV39H2	−1.0042614	0.785232355	2,121	2,112	unchanged
DNMT3A	DNA (cytosine-5)-methyltransferase 3A	−1.0125589	0.979807318	645	637	unchanged
MTR	Methionine synthase	−1.0139373	0.98656539	582	574	unchanged
*AHCYL2*	*Adenosylhomocysteinase*	*−1.0439059*	*0.002982796*	*5,944*	*5,694*	*downregulated*
*DOT1L*	*Histone-lysine N-methyltransferase,H3 lysine-79 specific*	*−1.0609936*	*0.040837852*	*2,157*	*2,033*	*downregulated*
*TCL1B*	*T-cell leukemia/lymphoma 1B*	*−1.1037151*	*0*	*15,835*	*14,347*	*downregulated*
*DNMT1*	*DNA (cytosine-5)-methyltransferase 1*	*−1.118876*	*0*	*18,476*	*16,513*	*downregulated*
*DNMT3B*	*DNA (cytosine-5)-methyltransferase 3B*	*−1.1300284*	*2.04E-06*	*2,781*	*2,461*	*downregulated*
*SUV39H1*	*Histone-lysine N-methyltransferase SUV39H1*	*−1.1617152*	*9.60E-13*	*4,145*	*3,568*	*downregulated*

*When the ratio is less than 1, it is converted to its negative inverse.

The transcriptome data have been deposited in the DDBJ Sequence Read Archive (DRA) with accession number DRA006403.

Bold show upregulated. Italic show downregulated.

### Gene expression pattern in granulosa cells

The rate-limiting enzymes in the glycolytic system (*HK2*, *ADPGK*, *PFKL*, *PFKM*, *PFKP*, *ALDOA*, *ALDOC*, and *PKM2*) and *SLC2A* genes were significantly upregulated in somatic cells surrounding oocytes (granulosa cells) following FSK and IBMX treatment (Supplementary Fig. 1, Table 5), similarly to the case of oocytes. On the other hand, the expression of genes related to mitochondrial electron transport system following FSK and IBMX treatment showed no clear uniform pattern and genes related to fatty acid degradation were mostly downregulated, unlike the case of oocytes (Supplementary Figs 2 and 3). The expression of genes related to ovarian steroidogenesis in granulosa cells was significantly upregulated following FSK and IBMX treatment (Supplementary Fig. 4), showing an increase of uptake of LDL and HDL into cells and steroidogenesis, such as 17β-estradiol to promote oocyte maturation.

**Table 5:** Comparison of gene expression pattern following an increase of cAMP levels in granulosa cells with that in previous reports.

Feature ID	Annotations - database object name	Khan *et al*., 2015	Present data	Experiment – fold change* (original values)	Kal’s Z-test: P-value correction	Number of mapped reads before FSK and IBMX treatment	Number of mapped reads after FSK and IBMX treatment
HK2	Protein-histidine pros-kinase	**upregulated**	**upregulated**	13.31469649	0	626	8,335
SLC2A13	Solute carrier family 2, facilitated glucose transporter member 13	not assessed	**upregulated**	3.9333333	**3.05E-09**	15	59
SLC2A1	Solute carrier family 2, facilitated glucose transporter member 1	**upregulated**	**upregulated**	3.9316843	**0**	3,396	13,352
SLC2A4	Solute carrier family 2, facilitated glucose transporter member 4	not assessed	unchanged	3	0.149116134	2	6
SLC2A3	Solute carrier family 2, facilitated glucose transporter member 3	unchanged	**upregulated**	2.8121891	**0**	19,903	55,971
SLC2A8	Solute carrier family 2, facilitated glucose transporter member 8	not assessed	**upregulated**	2.6663466	0	2,083	5,554
FSHR	Follicle-stimulating hormone receptor	unchanged	**upregulated**	2.59792	0	1,925	5,001
PGR	Progesterone receptor	**upregulated**	**upregulated**	2.58333	1.35E-15	60	155
SLC2A12	Solute carrier family 2, facilitated glucose transporter member 12	not assessed	**upregulated**	2.4375	**6.86E-08**	32	78
SLC2A10	Solute carrier family 2, facilitated glucose transporter member 10	not assessed	**upregulated**	2.3035714	**1.59E-11**	56	129
NR4A1	Nuclear receptor subfamily 4 group A member 1	**upregulated**	**upregulated**	2.19731	0	10,471	23,008
AKT2	v-Akt murine thymoma viral oncogene homologue 2	down	**upregulated**	1.47771	0	875	1,293
TP53	Cellular tumor antigen p53	*downregulated*	**upregulated**	1.33214	0	557	742
PDGFB	PDGFB protein	unchanged	unchanged	1.08451	0.122965	71	77
SLC2A2	Solute carrier family 2, facilitated glucose transporter member 2	not assessed	unchanged	1	1	0	0
AREG	Amphiregulin	**upregulated**	unchanged	−1	1	1	1
MAP2K3	Mitogen-activated protein kinase 3	**upregulated**	*downregulated*	−1.3971	0.000806	2,255	1,614
EGR1	Early growth response protein 1	*downregulated*	*downregulated*	−1.64276228	0	25,977	15,813
HAS2	Hyaluronan synthase 2	**upregulated**	unchanged	−1.8	0.10881	72	40
CCK	Cholecystokinin	**upregulated**	unchanged	−1.8333	0.447261	22	12
DUSP1	Dual-specificity protein phosphatase	*downregulated*	*downregulated*	−3.523	0	2,371	673
ADCYAP1	Pituitary adenylate cyclase-activating polypeptide	unchanged	*downregulated*	−3.5385	0.001078	46	13
GFPT2	Glutamine-fructose-6-phosphate transaminase 2	**upregulated**	*downregulated*	−4.54113924	0	1,435	316
STAR	Steroidogenic acute regulatory protein, mitochondrial	**upregulated**	*downregulated*	−37.4455	0	11,346	303

*When the ratio is less than 1, it is converted to its negative inverse.

The transcriptome data have been deposited in the DDBJ Sequence Read Archive (DRA) with accession number DRA006403.

Bold show upregulated. Italic show downregulated.

### cAMP response genes in bovine granulosa cells

Several genes have been shown to be involved in cAMP signaling in bovine granulosa cells^17^. The expression patterns of these genes following FSK and IBMX treatment were compared. The expression patterns of 7 of the 18 genes characterized as being involved in cAMP signaling were similar to those previously reported^17^ (Table 5). The differences for the other genes were probably due to short-term treatment (2 h). Although enzymes involved in cleavage of the EGF receptor-extracellular domain (*ADAMTS1*, *ADAM17*) were upregulated, the expression of EGF-like peptides was downregulated or unchanged, except for betacellulin. As a result, most genes involved in the signaling pathway of ErbB family proteins, which features four receptor tyrosine kinases and is structurally related to the epidermal growth factor receptor (*EGFR*), were downregulated or unchanged, except for tyrosine-protein kinase Src (*SRC*) and phosphatidylinositol 4,5-bisphosphate 3-kinase catalytic subunit alpha isoform (*PIK3CA*) (Supplementary Table).

## Discussion

The present study reveals that FSK and IBMX treatment at the prophase stage induced the expression of genes involved in glycolysis, fatty acid degradation, and mitochondrial functions in oocytes, and accordingly improved mitochondrial functions. Meiotic resumption has been shown to be inhibited by maintenance of the intracellular cAMP level in oocytes^4,5^. Although meiotic maturation was arrested during FSK and IBMX treatment (Fig. 1b), several genes regulating the cell cycle, such as cyclin-dependent kinase 1 (*CDK1*), were upregulated. Previous studies have shown that the expression of *CDK1*^18^, *CCNB2*, *PTTG1*, and *KS1B*^19^ increased in oocytes obtained from follicles larger than 6 mm compared with the levels in those smaller than 5 mm, showing that FSK and IBMX treatment promotes the accumulation of mRNAs in oocytes in the growing follicle stage, while maintaining meiotic arrest. In addition, histone *H2A* variants, *H2AFY* and *H2AFY2* (*macroH2A*), were upregulated in oocytes after FSK and IBMX treatment. *MacroH2A* associates with the heterochromatin of inactive X chromosomes in female somatic cells^20^ and can inhibit transcription because it can downregulate NF-κβ transcription factor binding and impedes SWI/SNF chromatin remodeling^21^, leading to transcriptional repression of genes in mature oocytes.

Mitochondria are directly involved in cellular energetic metabolism, growth, homeostasis, and death^22,23^. The production of ATP by oxidative phosphorylation is a well-known mitochondrial function, which is supported by respiratory chain complexes located on the inner membranes (Fig. 2a). NADH dehydrogenase oxidizes NADH produced through several catabolic pathways, such as glycolysis, pyruvate dehydrogenase, the tricarboxylic acid (TCA) cycle, and beta-oxidation of fatty acids. Succinate dehydrogenase oxidizes FADH_2_ supplied by the TCA cycle and beta-oxidation. The present study shows that the expression of genes involved in beta-oxidation and glycolysis was upregulated following FSK and IBMX treatment (Figs 3a and 4). Accordingly, NADH and FADH_2_ might be supplied by beta-oxidation and glycolysis. It has been shown that beta-oxidation is essential for mouse oocyte developmental competence and early embryo development^24,25^.

A study using *LMF1*-deficient mice demonstrated that endothelial lipase activity was diminished in the absence of *LMF1*^26^. Although lipase proteins were expressed at their normal levels in *LMF1*-deficient cells, they remained inactive^27^. Thus, lipase maturation critically depends on the client-specific chaperones residing in ER membranes, *LMFs*^28^. Although the expression of lipase remained unchanged, the expression of *LMFs* was upregulated. Accordingly, lipase activity would be increased. Moreover, *SCARB1*, low-density lipoprotein receptor (*LDLR*), and *FABP3* in oocytes and surrounding somatic cells would collaborate to supply an adequate amount of fatty acids for beta-oxidation in oocytes.

Electrons generated from NADH and FADH_2_, which are supplied from glycolysis and lipolysis, are transferred to ubiquinone at mitochondrial inner membrane. Reduced ubiquinone transfers electrons to cytochrome C reductase, which reduces cytochrome C. Then, cytochrome C transfers the electrons to cytochrome C oxidase, which produces H_2_O by consuming oxygen. The increases of oxygen consumption in mitochondria and mitochondria with active cytochrome C oxidase in oocytes (Fig. 2) indicated upregulation of the mitochondrial electron transport system following FSK and IBMX treatment.

Protons expelled to the intermembrane space by NADH dehydrogenase and cytochrome C reductase and oxidase generate protonic gradients. ATP synthase provides a channel for the entrance of protons into the mitochondrial matrix and the exergonic protonic flux drives the phosphorylation of ADP to ATP. As a result, ATP levels in oocytes increased following FSK and IBMX treatment (Fig. 2e).

Methionine adenosyltransferase (*MAT*), which was upregulated following FSK and IBMX treatment (Table 4), is a family of enzymes that catalyzes the synthesis of S-adenosylmethionine (SAM) from ATP and methionine^29^. *MAT*-mediated SAM synthesis is the first step in the methionine cycle, playing a central role in trans-sulfuration, polyamine synthesis, and transmethylation pathways^30^. SAM is the universal methyl donor for the epigenetic methylation of DNA and histones because it is the immediate precursor for methylation^31^. SAM is converted to S-adenosylhomocysteine (SAH). However, *AHCYL* by which *SAH* is hydrolyzed to homocysteine was downregulated in oocytes after FSK and IBMX treatment. Furthermore, most of the genes involved in the methylation of DNA and histone proteins were downregulated after the treatment. Taking these findings together, the synthesis of SAM might be initiated during the early phase of FSH stimulation, but the methylation of DNA and histones is not. Thus, FSK and IBMX treatment would permit greater accumulation of mRNAs that play an essential role in further development.

The rise in cAMP in granulosa cells has been implicated in the promotion of oocyte maturation. During *in vivo* maturation, a preovulatory luteinizing hormone surge causes a sharp rise in cAMP concentration in the mural granulosa cells^32^. As a result, cAMP induces the transient expression of the epidermal growth factor (EGF)-like peptides amphiregulin (*AREG*), epiregulin, and betacellulin, which are key paracrine mediators of granulosa cell expansion, oocyte maturation, and ovulation^33^. The secretion of EGF-like peptides prompts the activation of EGF receptors and subsequently leads to the phosphorylation of ERK1/2 and MAPK14 (p38)^32,34–36^. The activation of ERK1/2 is the key pathway to induce oocyte maturation following the EGF pathway because inhibition of the EGF receptor inhibits cumulus granulosa cell expansion, oocyte maturation, and ovulation^37,38^. The present work shows that most EGF-like peptides in cumulus granulosa cells were downregulated following FSK and IBMX treatment probably due to short-term treatment (2 h). Although the expression patterns of 7 of the 18 genes characterized as being involved in cAMP signaling were similar to those previously reported^17^ (Table 5), other 11 genes were different because the gene expression in granulosa cells was examined in cells treated with FSK and IBMX for 5 h^17^. When the granulosa cells were sampled 6 h after the start of maturation culture^39^, the expression pattern of genes related EGF pathway was also different from our data using granulosa cells immediately after FSK and BMX treatment for 2 h. In the present study, most genes involved in the signaling pathway of ErbB family proteins were also downregulated or unchanged. Thus, FSK and IBMX treatment would delay the process of cumulus granulosa cell expansion toward germinal vesicle breakdown in oocytes, as suggested by previous reports^17,40,41^. Simultaneously, the resumption of meiotic maturation of oocytes was also inhibited during FSK and IBMX treatment.

Consequently, the findings of this study indicate that FSK and IBMX treatment drastically changes gene expression patterns without involving oocyte nuclear maturation. Additionally, it has been shown that changes in the gene expression of granulosa cells are associated with an increase of developmental competence of bovine oocytes^42^. Taking these findings together, the gene expression of granulosa cells would change to support the developmental competence of oocytes without involving meiotic maturation.

Overall, the present study suggests the importance of a comprehensive energy-supply system in oocytes to support their development and reveals that the improvement of mitochondrial function under FSH stimulation is a potential explanation of how oocytes acquire developmental competence.

## Methods

### Oocyte collection and pre-maturation treatment

Bovine ovaries were obtained from Japanese Black cows and heifers at a local slaughterhouse in Osaka city and were transported to the laboratory in saline for 3–5 h at 18–21 °C. Bovine oocytes were collected from follicles (diameter, 2–5 mm), and oocytes with intact cumulus granulosa cells and evenly granulated cytoplasm (COCs) were selected and randomly assigned to each treatment. COCs were retrieved by follicle aspiration using 21-gauge needle.

COCs were exposed during pre-IVM to the adenylate cyclase activator FSK (F6886; Merck Millipore Co., Darmstadt, Germany) at 100 µM, which potently increases whole COC and intraoocyte cAMP levels, as well as to 500 µM IBMX (I5879; Merck Millipore Co.), a non-specific PDE inhibitor. COCs were maintained in pre-IVM treatments by exposing to both FSK and IBMX together in undermentioned IVM medium at 39 °C under 5% CO_2_ in air with high humidity for 2 h. At the end of the pre-IVM phase, COCs were washed twice in IVM medium, before transfer to fresh IVM medium. The above-described pre-IVM treatment has been shown to be effective measures to improve the oocyte developmental competence in cattle^39^. Control COCs were submitted to IVM immediately without meiotic inhibition.

Both sock solutions of 50 mM FSK and 10 mM IBMX were prepared in DMSO (D2650; Merck Millipore Co.). Thus, pre-IVM medium with FSK and IBMX contained 2% DMSO. To assess the effect of DMSO during pre-IVM treatment, bovine COCs were also cultured in pre-IVM medium contained 2% DMSO without FSK nor IBMX for measurement of OCRs.

### IVM

COCs were washed in IVM medium, which consisted of TCM 199 (Gibco, Grand Island, NY), 0.02 mg/ml FSH (Antrin; Kyoritsu Tokyo, Japan), 1 μg/ml estradiol-17β (E-8875; Merck Millipore Co.), 3 mg/ml BSA (A-4378; Merck Millipore Co.), and 1% (w/v) gentamycin solution (G1397; Merck Millipore Co.)^43^. Each group of 10 COCs was introduced into a 50-μl droplet of IVM medium in a plastic dish covered with mineral oil (M-8416; Merck Millipore Co.) immediately after oocyte collection (control) or following FSK and IBMX treatment. The COCs were cultured for 24 h at 39 °C under 5% CO_2_ in air with high humidity. During IVM, 20 oocytes per treatment from 3 independent experiments were fixed at several time points and stained to assess the time-dependent change of nuclear maturation. A total of 1080 oocytes were examined.

### *In vitro* fertilization (IVF) and culture

IVF and culture were performed as described previously^43^. Briefly, frozen-thawed spermatozoa were washed with a discontinuous Percoll solution (P1644; Merck Millipore Co.). Matured oocytes were inseminated with the washed spermatozoa (1 × 10^6^ cells/ml) in a glucose-free defined medium supplemented with 2 μg/ml of heparin (H-3393; Merck Millipore Co.). Oocytes were then completely freed from attached spermatozoa using vortex agitation 6 h after IVF, and some of them were fixed to assess fertilization. IVF was performed at 39 °C under 5% CO_2_ in air at high humidity. Denuded ova were cultured in bicarbonate-buffered synthetic oviduct fluid with amino acids supplemented with 3 mg/ml of BSA (A4378; Merck Millipore Co.), and 1% (w/v) antibiotic-mycotic solution (SOFaa)^43^. Then, 20 of these ova were placed in 50 μl of SOFaa and cultured under 5% CO_2_, 5% O_2_, and 90% N_2_ with high humidity. Development to the blastocyst stage was examined under a stereomicroscope (100×) at 168 h (Day 7) and 192 h (day 8) after IVF. The number of cells per blastocyst was estimated as previously described^44^. Briefly, 192 h after IVF, the embryos were immersed in fixative solution of ethanol with 25 μg/ml Hoechst 33342 (346–07951; Dojindo) overnight. Samples were mounted on a slide, and then examined under confocal microscopy to count the number of cells.

### cAMP contents of oocytes

Changes in cAMP in oocytes were measured using a homogeneous, bioluminescent, and high-throughput assay (cAMP-Glo, V1501; Promega Corporation, Madison, WI, USA), in accordance with the manufacturer’s instructions. The assay is based on the principle that cAMP stimulates protein kinase A holoenzyme activity, decreasing available ATP and leading to decreased light production in a coupled luciferase reaction. In brief, oocytes with (n = 120) or without exposure to FSK and IBMX (n = 400) were sampled in 20 μl of induction buffer after the removal of granulosa cells and were kept at −70 °C until assaying. The buffer was shaken for 15 min at room temperature to dissolve the sample before being developed with the detection buffer and substrate supplied with the Kinase-GloR Reagent. Finally, the luminescent signal was measured by a luminometer (Spectra Max GEMINI; Molecular Devices LLC, San Jose, CA, USA). FSK was used as a positive control.

### Transcriptome analyses

A total of 458 COCs were exposed to FSK and IBMX. Total RNA was extracted using the RNAqueous-Micro Kit (Thermo Fisher Scientific, Foster City, CA, USA) from oocytes or granulosa cells separately after the isolation of these cells. Granulosa cells were removed from COCs by vortexing and were recovered after centrifugation of cell suspension. The RNA of oocytes and granulosa cells without exposure to FSK and IBMX was also extracted from 491 COCs as a reference. Samples were obtained from 9 trials (approximately 50 COCs per trial). All samples were gathered together before RNA isolation. A total of granulosa cells was estimated at 9 × 10^6^ cells^43^.

Quality was checked using a 2100 BioAnalyzer (Agilent Tecnologies, Palo Alto, CA, USA) with RNA nano kit. After quality check, cDNA library was prepared using the TruSeq RNA Library Preparation Kit V2 (Illumina, San Diego, CA, USA). Derived cDNA libraries were checked for quality and quantity using a 2100 BioAnalyzer with DNA1000 kit. Quantitive PCR was also conducted to examine the quantity of the libraries using KAPA Library Quantification kit (KAPA Biosystems, Wilmington, MA, USA). Cluster generation on cBot (Illumina) was carried out for each library, and sequencing was performed on a HiSeq2500 as 50-bp single end reads. The cluster generation was conducted three times per sample. Two lanes for the four libraries were used for sequencing. CASAVA software ver.1.8.3 was used for image analysis, base calling, and quality filtering following the manufacturer’s instructions. CLC genomics workbench (Qiagen, Redwood City, CA, USA) was used for the further analysis. After filtering of the adapter sequence, ambiguous nucleotides (N), and low quality, the remained sequences were used for alignment to the bovine genome sequence (UMD3). The number of aligned sequence reads in each gene were counted and expression value were calculated. Statistical analysis was also performed to identify the differentially expressed genes. Upstream regulators of the significantly differentially expressed genes were predicted using Ingenuity Pathway Analysis (IPA; Qiagen). The expression levels of genes of interest related to cAMP signaling, glycolysis, fatty acid metabolism, and the electron transport chain (ETC) were compared between oocytes and granulosa cells with and without FSK and IBMX treatment. Information on target genes involved in glycolysis, fatty acid degradation, and ETC in cattle was obtained from the Kyoto Encyclopedia of Genes and Genomes database (map No. 0010, 00071, 0020). The transcriptome data have been deposited in the DDBJ Sequence Read Archive (DRA) with accession number DRA006403.

### OCR

The OCR of oocytes was measured using mitochondrial toxins. Either carbonyl cyanide-p-trifluoromethoxyphenylhydrazone (FCCP; C2920, Merck Millipore Co.) or sodium cyanide (380970; Merck Millipore Co.) was added to give final concentrations of 1 μM and 1 mM, respectively during the measurement of OCR by oocytes in the respiration buffer: human tubal fluid buffered with 21 mM HEPES containing 0.33 mM pyruvate and 2.7 mM glucose (Modified HTF Medium; Irvine Scientific, Santa Ana, CA, USA) supplemented with 5% Serum Substitute Supplement (SSS; IS Japan, Tokyo, Japan) (v/v). The concentration of FCCP and measuring condition were determined as described in Supplementary Fig. 5. Cyanide at 1 mM effectively blocks mitochondrial cytochrome oxidase activity. The OCR of oocytes was determined using scanning electrochemical microscopy (SCEM; CRAS1.0; Clino Ltd., Sendai, Japan) at 37 °C, as previously described^45^. In brief, each oocyte was moved into a well filled with the respiration buffer. The oocyte sank to the bottom of the cone-shaped microwell and remained at its lowest position. A platinum microdisk electrode was loaded into 5 mL of respiration buffer, and the tip potential was controlled at −0.6 V versus Ag/AgCl with a potentiostat to scan the local oxygen concentration. The microelectrode scanned along the z-axis from the edge of the sample, and the OCR was determined with custom software based on the spherical diffusion theory. The OCR was measured at three random points around the sample. Immediately after the measurement of OCRs of bovine oocytes in the respiration buffer, the samples were transferred into respiration buffer containing 1 μM FCCP. The OCRs of the sample in 1 μM FCCP was measured 30 min after the transfer and immediately moved into the buffer containing 1 mM cyanide. The OCRs of the sample in 1 mM cyanide was measured 10 min after the transfer. Mitochondrial OCR was calculated by subtracting the values obtained in the presence of cyanide from those obtained without FCCP or cyanide. The maximum mitochondrial OCR was calculated by subtracting the values obtained in the presence of cyanide from those obtained in the presence of FCCP. A total of 60 oocytes were used for OCR measurement in four independent runs.

### Determination for mtDNA copy number

All specimens were sampled individually in 2 μl DNase-free water and cryopreserved at −75 °C until assay. Each sample was lysed in 4 μl lysis buffer (0.4 mg/ml proteinase K (# P2308, Merck Millipore Co.), 20 mM Tris (#252859, Merck Millipore Co.), 0.9% Nonidet P-40 (# 213277-2, Merck Millipore Co.), and 0.9% Tween 20 (# P1379, Merck Millipore Co.)) at 55 °C for 30 min, followed by heating at 95 °C for 5 min. The lysate was diluted in DNase free water to a final volume of 40 μl. A 4-μl aliquot of the lysate was added to 12.5 μl Quantifast SYBR Green master mix (Qiagen, Venlo, Limburg, the Netherlands), 8.5 μl DNase-free water and 1 μM of each primer (Forward primer: 5-ATTTACAGCAATATGCGCCC-3 and Reverse primer: 5-AAAAGGCGTGGGTACAGATG-3). PCR amplifications was run in triplicate in a Rotorgene Q thermal cycler (Qiagen) according to the following conditions: 95 °C for 5 min, 40 cycles of 95 °C for 5 s, and 60 °C for 10 s. SYBR green fluorescence was measured at the end of each cycle. The melting curve was analyzed to check the specificity of the PCR products. A standard curve was prepared for each run using tenfold serial dilutions representing copies of the external standard (10^3^ to 10^6^ copies). The external standard used in the present study was the PCR product of the corresponding gene cloned into a vector using the Zero Blunt TOPO PCR cloning kit (Invitrogen Life Sciences, Carlsbad, CA, USA), and the product was confirmed by sequencing before use. The primers were designed using Primer3Plus (http:// sourceforge.net/projects/primer3/) and ND5 (1.82 kb region from 12,109 to 13,929) of bovine mitochondria (NC_006853.1).

### Mitochondrial function and lipid content determined using confocal laser microscopy (CLM)

COCs were denuded by vortexing for 10 min. Denuded oocytes were incubated with 0.5 μM MitoTracker Orange CM-H2TMRos (M-7511; Life Technology, Carlsbad, CA, USA) for 0.5 h in bicarbonate-buffered TCM199 supplemented with 0.1% poly(vinyl alcohol) (PVA-199; Merck Millipore Co.) for 20 min at 39 °C under 5% CO_2_, 5% O_2_, and 90% N_2_ and then washed three times in PVA-199. Stained oocytes were transferred into a 3-μL droplet of PVA-199 on a glass-bottomed culture dish (P35G-0-14-C; MatTek Corporation, Ashland, MA, USA). Microscopic images were obtained using a CLM (CellVoyager CV1000; Yokogawa Electronic, Tokyo, Japan) at 40× and 39 °C under 5% CO_2_, 5% O_2_, and 90% N_2_ and fluorescence intensity in the equatorial plane of oocytes except for the nuclear region was measured using ImageJ (http://imagej.nih.gov/ij/). In this study, a total of 120 COCs were used for the analysis of mitochondrial activity in three independent runs.

Denuded oocytes were then washed again in PBS containing 0.1% PVA (PVA-PBS), fixed in 2% (v/v) paraformaldehyde (167–25981; Wako Chemicals, Osaka, Japan) at 37 °C for 30 min, and stored in PVA-PBS at 4 °C for a maximum of 1 week. Oocytes were washed twice in PVA-PBS, permeabilized for 30 min in PVA-PBS with 0.1% (v/v) Triton X-100 (Merck Millipore Co.), and washed in PVA-PBS. To assess the maturational stage, oocytes were stained with 10 μg/ml Hoechst 33342 (346–07951; Dojindo, Kumamoto, Japan) for 30 min and subsequently washed three times in PVA-PBS. Neutral lipids in lipid droplets were stained in accordance with a previous report^46^. Lipid droplets were stained with the specific neutral lipid stain BODIPY™ 493/503 (20 μg/ml, 1 h, D3922; Molecular Probes, Eugene, OR, USA) in PVA-PBS, and oocytes were washed three times in PVA-PBS. Microscopic images were obtained at a magnification of 40× and fluorescence intensity in the equatorial plane of the oocytes was measured using ImageJ in a manner similar to that for MitoTracker staining. In this study, a total of 126 COCs were used for lipid droplet analysis in three independent runs.

### Cytochrome C oxidase activity and lipid content determined using transmission electron microscopy (TEM)

Oocytes were fixed to observe cytochrome C oxidase (CCO) activity, as described in a previous report^47^. Briefly, they were fixed in 2% (v/v) glutaraldehyde (TAAB Laboratories, Aldermaston, UK) and 0.15 M sucrose (Wako Chemicals) in 0.05 M PBS for 15 min at 4 °C. After rinsing in the same buffer, the oocytes were incubated in 1.4 mM 3,3′-diaminobenzidine (DAB) tetrahydrochloride (Merck Millipore Co.), 0.1% (w/v) cytochrome C type II (Sigma), 0.23 M sucrose, and 0.0002% catalase (w/v) (Merck Millipore Co.) in 0.05 M PBS for 3 h at 37 °C. Oocytes were then washed in PBS for 1 h. Subsequently, they were post-fixed in 1% (v/v) osmium tetroxide (TAAB Laboratories) for 2 h at 4 °C. Next, they were dehydrated in increasing concentrations of ethanol and embedded in epoxy resins. Ultrathin sections were stained with uranyl acetate and examined using TEM (JEM-1011; JEOL, Tokyo, Japan). The content of neutral lipids was estimated by calculating the surface ratio of neutral lipids per surface area of the oocyte excluding the surface area of the nucleus. The surface areas of each of neutral lipids, oocyte, and nucleus were measured using Digital Micrograph version 3.7.4 (Gatan, Pleasanton, CA, USA)^48^. In this study, a total of five oocytes were examined in each treatment.

### ATP in oocytes

The ATP level of oocytes was determined as luminescence emitted in an ATP-dependent luciferin–luciferase bioluminescence assay (ATP assay kit; Toyo-Inc., Tokyo, Japan). Serial dilutions of the ATP standard ranging from 100 nM to 0.01 pM were prepared, and a standard curve was prepared based on the relative light intensity of the serial dilution standard (log luminescence vs. log[ATP]). The standard curve was used to calculate the ATP level of the samples. Sample measurements were conducted in duplicate and the mean was calculated. For the preparation of each sample, one oocyte was added to 50 μL of distilled water. Oocyte was lysed and the luminescence was then measured immediately using a luminometer (Gene Light 55; Microtec, Funabashi, Japan). Ten oocytes per trial were sampled individually from three independent experiments. A total of 60 oocytes were used for the analysis.

### Statistical analysis

IPA was used to determine how many known targets of each transcription regulator were present in the list of differentially expressed genes, and a statistical test was performed to consider its significance. Fisher’s exact test was used for the statistical analysis of gene set enrichment in each functional category. Differences between two groups were analyzed using unpaired Student’s *t*-test or chi-square test for embryo development. When more than two groups were compared, analysis of variance (ANOVA) followed by the Tukey–Kramer test was used. Normality and homogeneity of variances were confirmed by Shapiro-Wilk and Levene test before parametric analyses (t-test and ANOVA) were run. The data are shown as the mean ± SD. A P value less than 0.05 was considered to be significant.

## Supplementary information

Supplementary data set

## Data Availability

The datasets generated during and/or analyzed during the current study are available from the corresponding author on reasonable request.
