# Supplementary material for: Mitochondrial function in immature bovine oocytes is improved by an increase of cellular cyclic AMP
Source: Sci Rep. 2019 Mar 26;9:5167. doi: 10.1038/s41598-019-41610-6 (PMC6435665; doi:10.1038/s41598-019-41610-6)
Supplement: Supplementary file 1 — Supplementary data set [file 41598_2019_41610_MOESM1_ESM.pdf]

# Title: Mitochondrial function in immature bovine oocytes is improved by an increase of cellular cyclic AMP

Authors: Shu Hashimoto[1*], Masaya Yamanaka[2], Takayuki Yamochi[1], Hisataka Iwata[3], Ryouka Kawahara-Miki[4], Masayasu Inoue[2], Yoshiharu Morimoto[5]

[1]Graduate School of Medicine, Osaka City University, Osaka 545-8585, Japan, [2]IVF Namba Clinic, Osaka 550-0015, Japan, [3]Department of Animal Science, Tokyo University of Agriculture, Kanagawa 243-0034, Japan, [4]Nodai Genome Research Center, Tokyo University of Agriculture, Tokyo 156-8502, Japan, [5]HORAC Grandfront Osaka Clinic, Osaka 530-0011, Japan
*Corresponding author. Reproductive Science, Graduate School of Medicine, Osaka City University, Osaka 545-8585, Japan

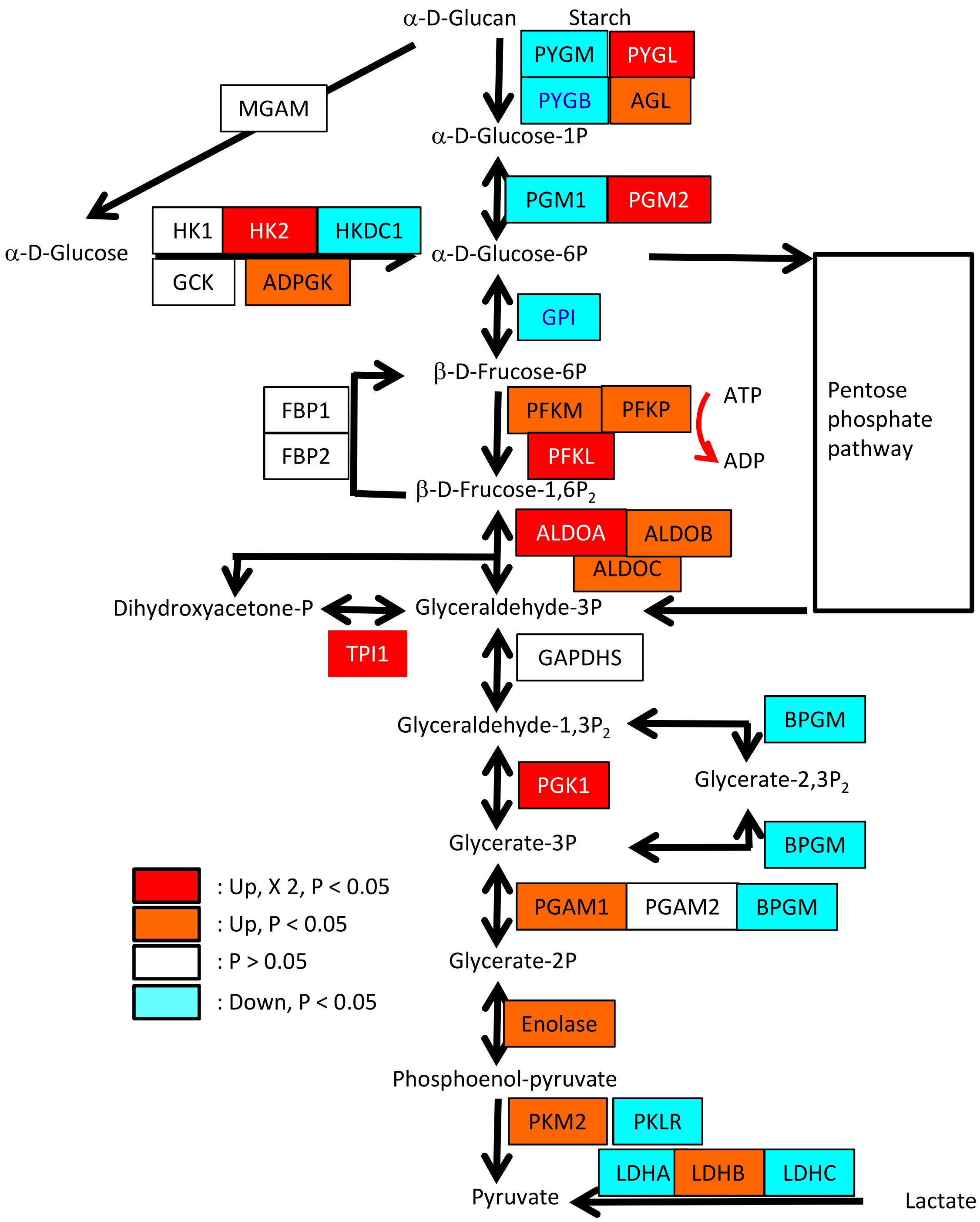


Supplementary Figure 1. Schema of expression of genes involved in glycolysis in granulosa cells following FSK and IBMX treatment. Each box shows enzymes involved in glycolysis. Red and orange boxes indicate that gene expression is significantly upregulated ($P < 0.05$). White and blue boxes show that gene expression is unchanged and downregulated, respectively. Red box also means that the expression level is increased more than double in granulosa cells after FSK and IBMX treatment compared with control granulosa cells. PYGL, PYGM, and PYGB: glycogen phosphorylases; AGL: glycogen debranching enzyme; MGAM: maltase-glucoamylase; PGM1: phosphoglucomutase-1; PGM2: phosphoglucomutase-2; HK1, HK2, and HKDC1: hexokinases; GCK: glucokinase; ADPGK: ADP-dependent glucokinase; GPI: glucose-6-phosphate isomerase;  PFKM, PFKL, and PFKP: 6-phosphofructokinases 1; FBP1 and FBP2: fructose-1,6-bisphosphatases I; ALDOA, ALDOB, and ALDOC: fructose-bisphosphate aldolases, class I; GAPDH: glyceraldehyde 3-phosphate dehydrogenase; BPGM: bisphosphoglycerate/phosphoglycerate mutase; PGK1: phosphoglycerate kinase; PGAM1 and PAM2: 2,3-bisphosphoglycerate-dependent phosphoglycerate mutases; TPI1: triosephosphate isomerase; PKM: pyruvate kinase; PKLR: pyruvate kinase isozymes R/L; LDHA, LDHB: lactate dehydrogenase. HK2, ADPGK, PFKL, PFKM, PFKP, ALDOA, ALDOC, and PKM2 are rate-limiting enzymes in the glycolytic system.

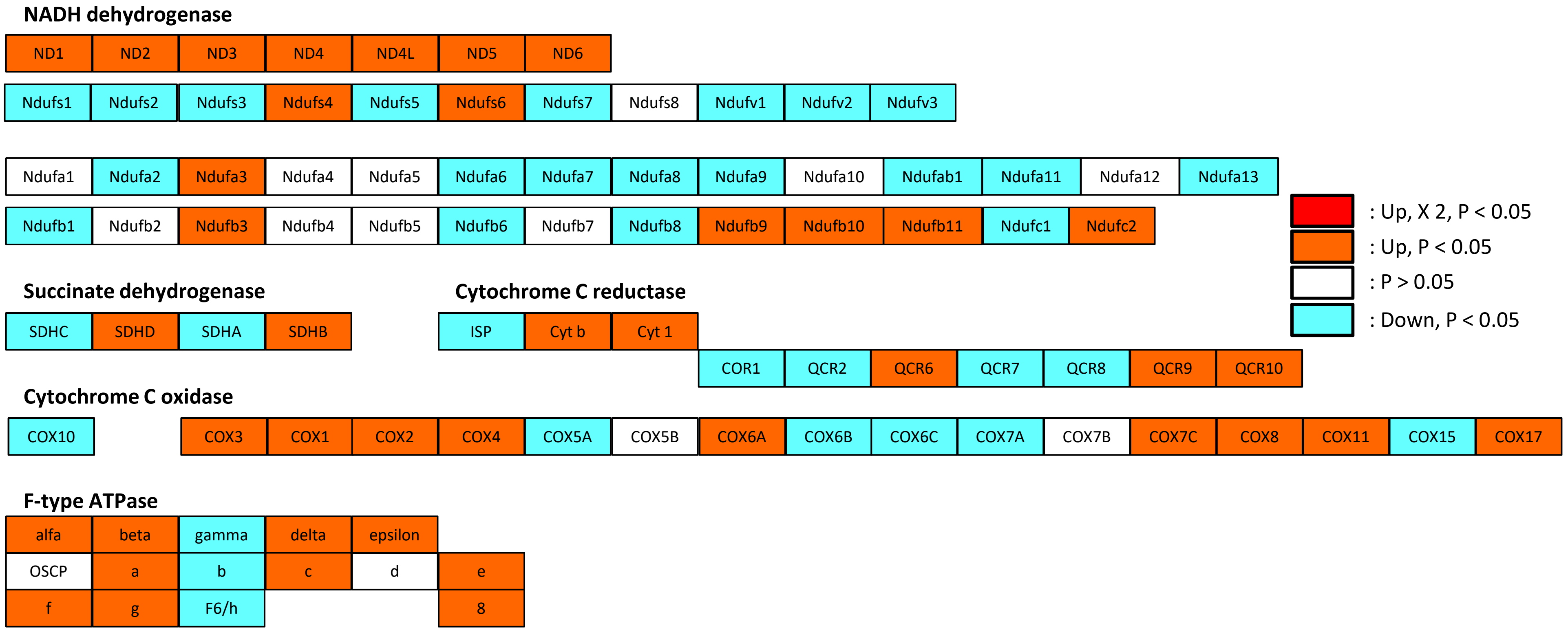


Supplementary Figure 2. Schema of mitochondrial complexes on inner mitochondrial membrane in granulosa cells . Each box shows protein constituents of mitochondrial complex, NADH dehydrogenase, succinate dehydrogenase, cytochrome C reductase, cytochrome C oxidase, and ATPase. Red and orange boxes indicate that gene expression is significantly upregulated ($P < 0.05$). White and blue boxes show that gene expression is unchanged and downregulated, respectively. Red box also means that the expression level is increased more than double in granulosa cells after FSK and IBMX treatment compared with control granulosa cells.

This schema was produced in reference to KEGG pathway for oxidative phosphorylation in Bos Taurus (https://www.genome.jp/kegg-bin/show_pathway?org_name=bta&mapno=00190&mapscale=&show_description=show).

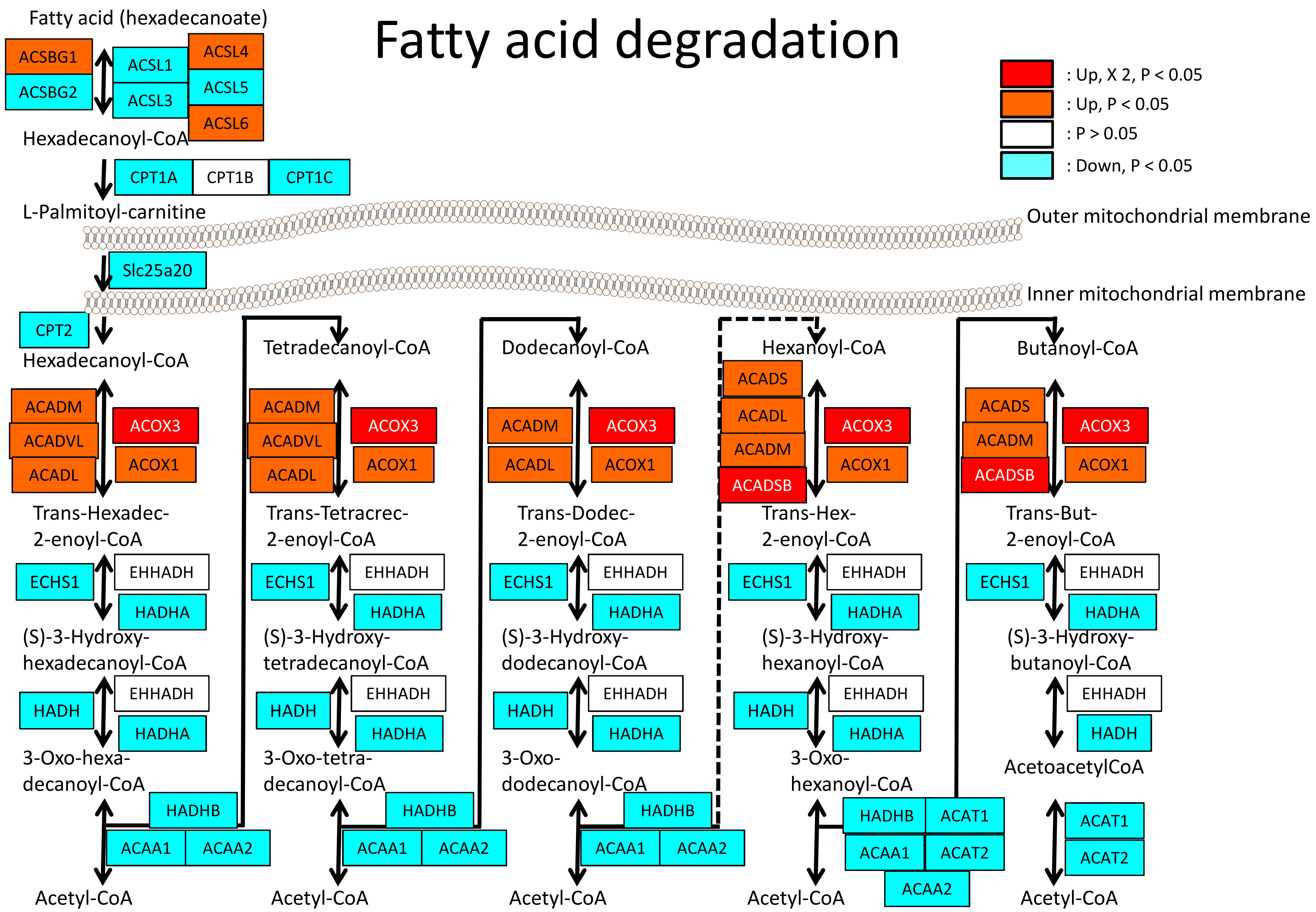


Supplementary Figure 3. Schema of beta oxidation in granulosa cells. Each box shows enzymes involved in beta-oxidation. Red and orange boxes indicate that gene expression is significantly upregulated ($P < 0.05$). White and blue boxes show that gene expression is unchanged and downregulated, respectively. Red box also means that the expression level is increased more than double in granulosa cells after FSK and IBMX treatment compared with control granulosa cells.

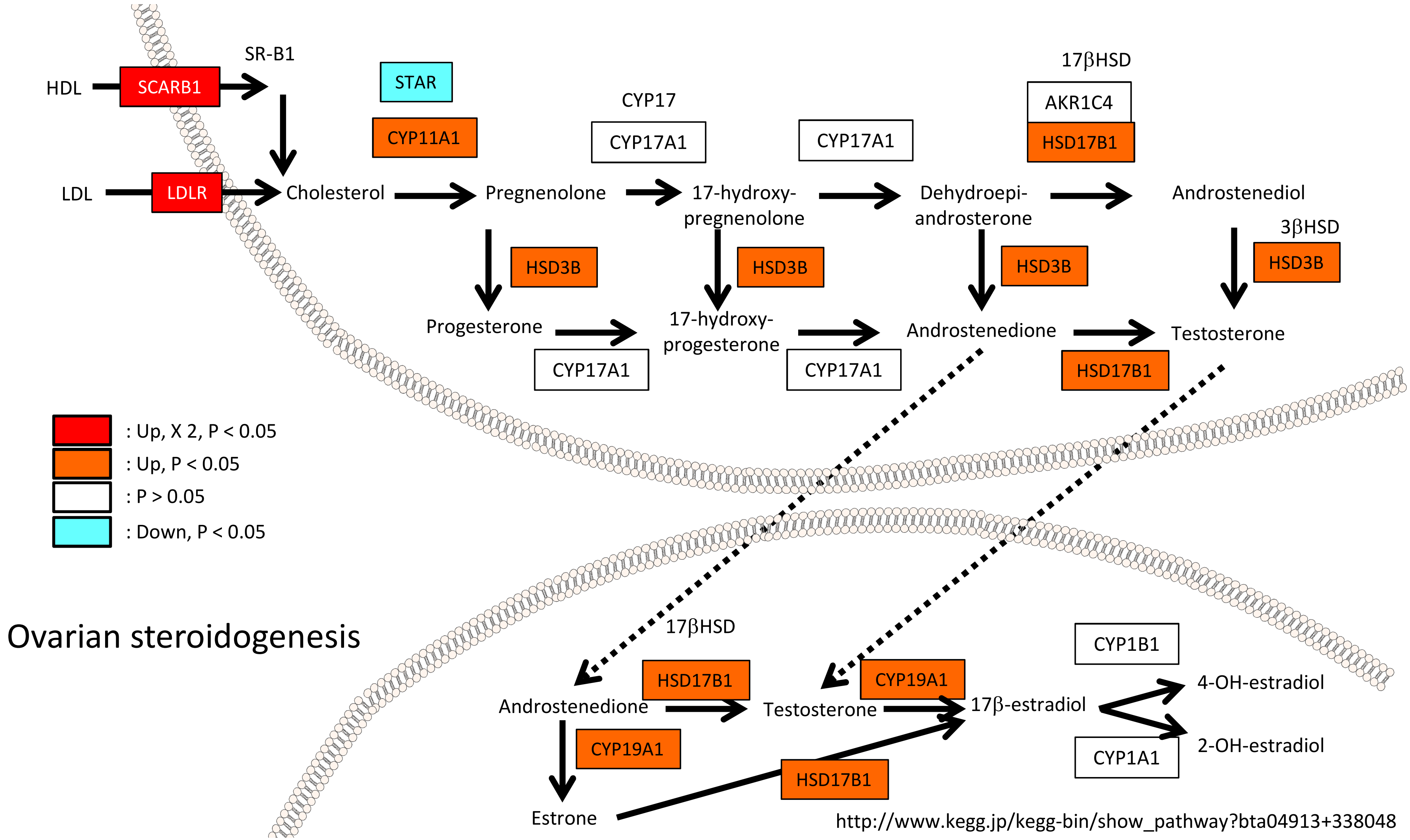


Supplementary Figure 4. Change of expression of genes involved in ovarian steroidogenesis in granulosa cells . Red and orange boxes indicate that gene expression is significantly upregulated ($P < 0.05$). White and blue boxes show that gene expression is unchanged and downregulated, respectively. Red box also means that the expression level is increased more than double in granulosa cells after FSK and IBMX treatment compared with control granulosa cells. The expression of genes related to ovarian steroidogenesis in granulosa cells was significantly upregulated following FSK and IBMX treatment. SCARB1: scavenger receptor class B member 1; LDLR: low-density lipoprotein receptor; STAR: steroidogenic acute regulatory protein; CYP11A1: cytochrome P450, family 11, subfamily A, polypeptide 1; CYP17A1: cytochrome P450, family 17, subfamily A, polypeptide 1; AKR1C4: aldo-keto reductase family 1, member C4; HSD17B1: hydroxysteroid 17-beta dehydrogenase 1; HSD3B: hydroxy-delta-5-steroid dehydrogenase, 3 beta- and steroid delta-isomerase 1; CYP19A1: cytochrome P450, family 19, subfamily A, polypeptide 1 (aromatase); CYP1A1: cytochrome P450, subfamily I (aromatic compound-inducible), polypeptide 1; CYP1B1: cytochrome P450, family 1, subfamily B, polypeptide 1.

Supplementary Table. Change of expression of genes involved in ErbB signaling pathway in somatic cells surrounding oocytes.

| Feature ID | Annotations - database object name | Experiment – fold change* (original values) | Kal's Z-test: P-value correction | Control | cAMP | |
|---|---|---|---|---|---|---|
| SRC | Tyrosine-protein kinase Src | 2.509901 | 0 | 404 | 1,014 | upregulated |
| BTC | Betacellulin | 2.282158 | 0 | 241 | 550 | upregulated |
| STAT5A | Signal transducer and activator of transcription | 1.833333 | 0 | 300 | 550 | upregulated |
| PIK3CA | Phosphatidylinositol 4,5-bisphosphate 3-kinase catalytic subunit alpha isoform | 1.557411 | 0 | 479 | 746 | upregulated |
| CBLC | E3 ubiquitin-protein ligase CBL | #DIV/0! | 0.43022 | 0 | 1 | unchanged |
| AREG | Amphiregulin | -1 | 1 | 1 | 1 | unchanged |
| GRB2 | Growth factor receptor-binding protein 2 | -1.04579 | 0.002566 | 708 | 677 | downregulated |
| PIK3CG | PIK3CG protein | #DIV/0! | 0.343036 | 2 | 0 | unchanged |
| PIK3CB | Phosphatidylinositol-4,5-bisphosphate 3-kinase | -1.08974 | 0.2471 | 255 | 234 | unchanged |
| PIK3R5 | Phosphoinositide-3-kinase, regulatory subunit | -1.12121 | 0.986831 | 37 | 33 | unchanged |
| PLCG1 | Phosphoinositide phospholipase C | -1.30913 | 0.095171 | 3,371 | 2,575 | unchanged |
| PIK3R2 | Phosphatidylinositol 3-kinase regulatory subunit beta | -1.38643 | 0.0168 | 1,410 | 1,017 | downregulated |
| PIK3R1 | Phosphatidylinositol 3-kinase regulatory subunit alpha | -1.77778 | 4.37E-09 | 784 | 441 | downregulated |
| CRK | Proto-oncogene C-crk | -2.01917 | 0 | 4,107 | 2,034 | downregulated |
| SHC1 | SHC-transforming protein 1 | -2.80526 | 0 | 10,444 | 3,723 | downregulated |
| STAT5B | Signal transducer and activator of transcription 5B | -3.22881 | 0 | 762 | 236 | downregulated |
| CBLB | E3 ubiquitin-protein ligase CBL | -5.7839 | 0 | 1,365 | 236 | downregulated |
| NCK1 | NCK adaptor protein 1 | -8.30496 | 0 | 4,684 | 564 | downregulated |
| NRG1 | neuregulin 1 | -9 | 0.052897 | 9 | 1 | unchanged |
| CBL | E3 ubiquitin-protein ligase CBL | -10.0533 | 0 | 1,699 | 169 | downregulated |
| NRG3 | Neuregulin 3 | -21.6 | 0 | 108 | 5 | downregulated |
| HBEGF | Heparin-binding EGF-like growth factor | -24.3163 | 0 | 7,149 | 294 | downregulated |
| NRG2 | Neuregulin 2 | -26.0652 | 0 | 1,199 | 46 | downregulated |
| PLCG2 | Phosphoinositide phospholipase C | -37.0351 | 0 | 2,111 | 57 | downregulated |
| PIK3CD | Phosphatidylinositol-4,5-bisphosphate 3-kinase | -55.875 | 0 | 447 | 8 | downregulated |

*When the ratio is less than 1, it is converted to its negative inverse.
The transcriptome data have been deposited in the DDBJ Sequence Read Archive (DRA) with accession number DRA006403.

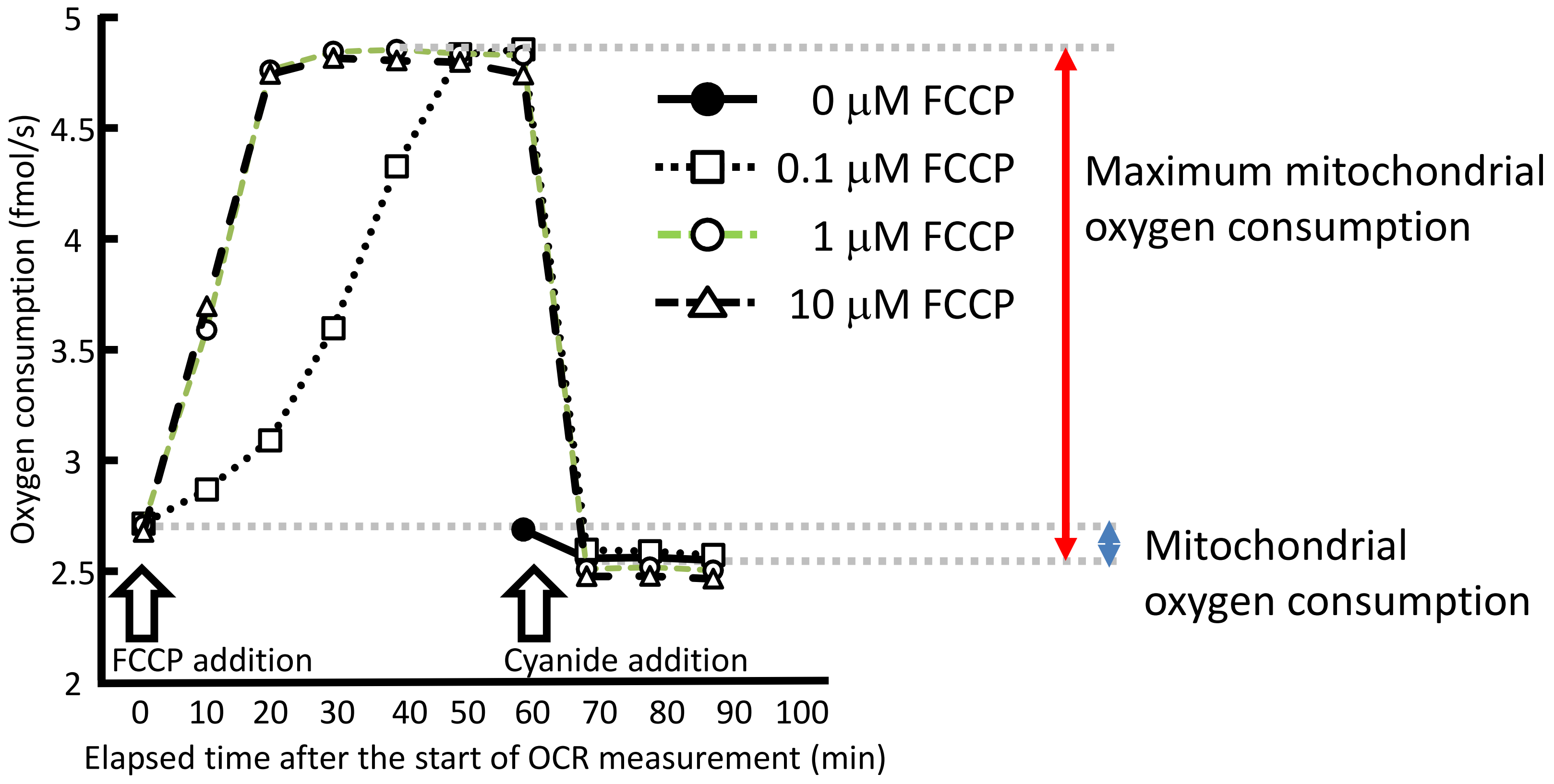


Supplementary Figure 5. The determination of measuring condition for OCRs. At first, the OCRs of bovine oocytes in the respiration buffer were measured. Immediately after the 1st OCR measurement, the samples were transferred into respiration buffer containing 0.1, or 1, 10 µM FCCP and moved into the buffer containing 1 mM cyanide 70 min after the start of OCR measurement. The measurement of the OCR was conducted every 10 min until 90 min. The OCRs of the sample in 1 or 10 µM FCCP sharply increased 10 min after the exposure to FCCP. On the other hand, the OCRs in 0.1 µM FCCP gradually increased until 50 min. The OCRs of all samples (0-10 µM FCCP) went down precipitously 10 min after the exposure to cyanide. Five oocytes were used in each treatment. From these data, we decided to measure the OCRs 30 min after the exposure to 1 µM FCCP and 10 min after the exposure to cyanide.
